# Tapping into rhythm generation circuitry in humans during simulated weightlessness conditions

**DOI:** 10.3389/fnsys.2015.00014

**Published:** 2015-02-18

**Authors:** Irina A. Solopova, Victor A. Selionov, Francesca Sylos-Labini, Victor S. Gurfinkel, Francesco Lacquaniti, Yuri P. Ivanenko

**Affiliations:** ^1^Laboratory of Neurobiology of Motor Control, Institute for Information Transmission Problems, Russian Academy of ScienceMoscow, Russia; ^2^Laboratory of Neuromotor Physiology, IRCCS Fondazione Santa LuciaRome, Italy; ^3^Centre of Space Bio-medicine, University of Rome Tor VergataRome, Italy; ^4^Biomedical Engineering Department, Oregon Health and Science UniversityPortland, OR, USA; ^5^Department of Systems Medicine, University of Rome Tor VergataRome, Italy

**Keywords:** central pattern generator, sensory input, rhythmogenesis, locomotion, humans

## Abstract

An ability to produce rhythmic activity is ubiquitous for locomotor pattern generation and modulation. The role that the rhythmogenesis capacity of the spinal cord plays in injured populations has become an area of interest and systematic investigation among researchers in recent years, despite its importance being long recognized by neurophysiologists and clinicians. Given that each individual interneuron, as a rule, receives a broad convergence of various supraspinal and sensory inputs and may contribute to a vast repertoire of motor actions, the importance of assessing the functional state of the spinal locomotor circuits becomes increasingly evident. Air-stepping can be used as a unique and important model for investigating human rhythmogenesis since its manifestation is largely facilitated by a reduction of external resistance. This article aims to provide a review on current issues related to the “locomotor” state and interactions between spinal and supraspinal influences on the central pattern generator (CPG) circuitry in humans, which may be important for developing gait rehabilitation strategies in individuals with spinal cord and brain injuries.

## Introduction

It is now largely accepted that the neural circuitry controlling locomotion involves a central pattern generator (CPG; Grillner, 1981). CPG functioning depends on supraspinal inputs and sensory feedback (Shik, 1997; Orlovsky et al., 1999; Pearson, 2004; Jordan et al., 2008). Most CPGs are quiescent under resting condition and become recruited by supraspinal pathways with command function (Grillner, 2006). Sensory activity establishes the timing of major phase transitions and contributes to the production of motoneuronal drive (Nielsen and Sinkjaer, 2002; Pearson, 2004), and may also trigger a stepping-like output (Sherrington, 1910; Gurfinkel et al., 1998; Gerasimenko et al., 2010).

The capacity of the mammalian lumbosacral spinal cord to generate rhythmic activity in the absence of input from the brain is firmly established in animal models (Sherrington, 1910; Graham Brown, 1912; Grillner, 1981) and there is indirect evidence that CPGs may also be a feature of the human spinal cord (Bussel et al., 1996; Minassian et al., 2004; Shapkova, 2004; Dominici et al., 2011; Hubli and Dietz, 2013; Ivanenko et al., 2013). The available evidence suggests that many locomotor-related movements that humans perform routinely (walking, running, cycling, swimming, crawling, backward walking, etc.) use similar rhythm circuitry but additionally require specialized control circuits (Zehr, 2005; Patrick et al., 2009; Hoogkamer et al., 2014). In fact, the capacity of neural circuits to generate rhythmic activity represents the common core for various locomotor tasks (Zehr, 2005). The aim of this article is to provide a review on current issues related to the excitability of spinal CPG circuitry in humans. Under normal conditions, it is sometimes difficult to investigate impairments in the CPG functioning due to interference with the ongoing task of body weight and balance control (including intense feedback). Therefore, one might examine the rhythmogenesis capacity of spinal circuitry in conditions not-complicated by these two factors.

Body weight support systems coupled with robotic devices or pharmacologic treatments are now often used in the rehabilitation practice to assist locomotor recovery in individuals with neuromotor disorders (Dietz, 2009; Sale et al., 2012; Hubli and Dietz, 2013; Valentin-Gudiol et al., 2013; Meyns et al., 2014; Moraru and Onose, 2014). There is still limited evidence of the efficacy of treadmill interventions with body weight support in some injured populations due to the complex nature of the control of locomotion, compensatory strategies, and plasticity of neuronal networks (Grasso et al., 2004; Picelli et al., 2013; Valentin-Gudiol et al., 2013; Swinnen et al., 2014; Sylos-Labini et al., 2014b). We will not review here any detailed analysis of clinical outcomes for ambulation when using locomotor training with body weight support systems and refer to other reviews (e.g., Wirz et al., 2005; Sale et al., 2012; Valentin-Gudiol et al., 2013; Scivoletto et al., 2014). The main focus here is to give emphasis to a facilitatory effect of simulated weightlessness on rhythmogenesis and its potential for assessing the state of the CPG circuits and for gait recovery after spinal cord injury and other neuromotor disorders.

## Locomotor “state” of the spinal circuity

Historically, Goltz and Freusberg (1874) were the first to report spontaneous air-stepping of the hindlimbs of the spinal dog before voiding the distended bladder, presumably due to some excitatory state of the spinal circuitry. In decerebrated animals exhibiting spontaneous fluctuations in their level of rigidity, rhythmic movements can be evoked by peripheral stimulation, provided there is an appropriate level of background extensor tonus and that the tonus is neither too low nor too high (Beritoff, 1915). In addition, an increase in tonus precedes the initiation of locomotion (Mori et al., 1982). The excitability status or state of the spinal network is thus of particular importance (Edgerton et al., 2008). Air-stepping can be used as a unique and important model for investigating human rhythmogenesis since its manifestation is largely facilitated by a reduction of external resistance, such as that resulting from body weight unloading (Gurfinkel et al., 1998; Selionov et al., 2009). Below we consider various experiments and observations in conditions of reduced gravity effects that help revealing the intrinsic properties of locomotor pattern generators and making evident the facilitation of non-voluntary limb stepping in humans.

**Figure 1:**
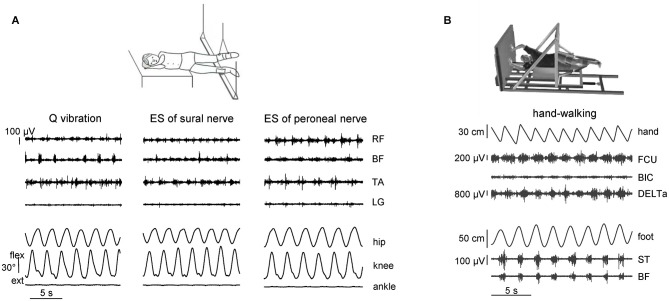
**Eliciting non-voluntary limb stepping movements in simulated weightlessness (gravity neutral) conditions. (A)** examples of non-voluntary rhythmic movements of the suspended legs induced by quadriceps (Q) muscle vibration and electrical stimulation (ES) of sural and peroneal nerves in one representative subject from the study of Selionov et al. (2009). An upward deflection of traces denotes flexion in the hip and knee joint angles and dorsiflexion in the ankle joint. Note the absence of ankle joint rotations during evoked air-stepping. **(B)** An example of evoked rhythmic leg movements during hand walking in one subject from the study of Sylos-Labini et al. (2014a). RF, rectus femoris, BF, biceps femoris, TA, tibialis anterior, LG, lateral gastrocnemius, FCU, flexor carpi ulnaris, BIC, biceps brachii, DELTa, anterior deltoid, ST, and semitendinosus. Hand and foot denote anterior-posterior displacements of the left hand and foot.

The spinal CPG circuitry can be activated in healthy humans by applying tonic central or peripheral sensory inputs. As we previously mentioned, in addition to the control of the timing of major phase transitions and muscle activity production (Nielsen and Sinkjaer, 2002; Pearson, 2004), sensory activity has access to the functional state of CPG and may initiate a stepping-like output (Sherrington, 1910; Gurfinkel et al., 1998; Gerasimenko et al., 2010). Figure 1A illustrates different examples of stimulation techniques that were explored for eliciting non-voluntary air-stepping: continuous muscle vibration (40–60 Hz, ~1 mm amplitude), and electrical stimulation of the superficial peroneal or sural nerves (0.3 ms duration pulses, 2–3 mA, 60 Hz) (Selionov et al., 2009). To minimize interference with the ongoing task of body weight and balance control, stepping movements are elicited during air-stepping in the absence of gravity influences and reduced external resistance. The subjects were tested while lying on their side with the legs supported using long ropes attached to the ceiling (Figure 1A) or using an exoskeleton (Figure 1B) so that they provided low-friction pendulum-like leg motion in the horizontal plane with a limited vertical motion component. The afferent signals due to vibration or electrical stimulation of peripheral nerves may increase the excitability of several segments of the spinal cord, which may facilitate triggering of locomotor-like movements. The latency of the elicited cyclic movements varied significantly across subjects and conditions (range 1–25 s). The delay in the onset of leg movement likely reflects the general property of the pattern generation circuitry and transition from tonic activation to the phasic CPG output. Generally, cyclic movements increased monotonically for 2–10 cycles until they reached a relatively constant amplitude of angular oscillations (Gurfinkel et al., 1998; Selionov et al., 2009; Gerasimenko et al., 2010). The characteristics of non-voluntary air-stepping (amplitude, cycle duration) were similar to the voluntary stepping in the same conditions.

In addition to peripheral sensory stimulation, central tonic facilitatory influences may be used for eliciting rhythmic leg movements, such as the Jendrassik maneuver and the Kohnstamm phenomenon (Gurfinkel et al., 1998; Selionov et al., 2009). An intriguing approach related to the role of tonic influences is the Kohnstamm phenomenon (Kohnstamm, 1915), which consists in the appearance of involuntary tonic activity and a particular sensation of “lightness” after the cessation of a long-lasting (30–40 s) isometric effort. Post-activation phenomena can therefore be used as a tool to study tonic influences. After-effects of a voluntary, long-lasting contraction in the leg muscles featured alternating rhythmic leg movements that lasted for about 20–40 s (Selionov et al., 2009), corresponding roughly to a typical duration of the post-contraction activity (Craske and Craske, 1986; Duclos et al., 2004; Ivanenko et al., 2006b). The difference in the effects of the two techniques (the post-contraction phenomenon and the Jendrassik maneuver) may point to the importance of tonic activation of the lumbosacral enlargement, since voluntary arm contractions (due to the Jendrassik maneuver) are weaker in evoking stepping movements: they act primarily on the cervical spinal cord and are not sufficient to evoke air-stepping unless the experimenter triggers them (Selionov et al., 2009).

Other techniques for triggering stepping movements are based on the more direct stimulation of the spinal cord by electromagnetic (Gerasimenko et al., 2010), transcutaneous or epidural electrical stimulation (Shapkova and Schomburg, 2001; Gorodnichev et al., 2012), which can initiate and sustain movements more robustly than by stimulation of sensory afferent fibers. Transcutaneous electrical spinal cord stimulation (at 5–40 Hz) is applied over T11-T12 vertebrae and presumably activates the locomotor circuitry through the dorsal roots (Gorodnichev et al., 2012; Gerasimenko et al., 2014), while epidural stimulation is based on an implanted array of electrodes directly placed over the back portion of the lower thoracic-upper lumbar spinal cord (Figure 4A, upper panel). Rhythmic locomotor-like leg movements in a gravity neutral position can be evoked in ~10–50% of healthy subjects, and the degree of activation may depend on supraspinal influences and the state and the rhythmogenesis capacity of the spinal circuitry (Gurfinkel et al., 1998; Selionov et al., 2009; Gerasimenko et al., 2010). The common feature of all stimulations described above is that they are tonic. In this respect, they corroborate earlier pioneering observations in decerebrate cats that stepping can be induced using a simple tonic stimulation pattern applied to the mesencephalic locomotor region (Shik et al., 1966), but they also show that this type of control can be initiated at the lumbosacral spinal cord level. Overall, the findings suggest that nonspecific tonic excitability may elicit or facilitate CPG activity.

**Figure 2:**
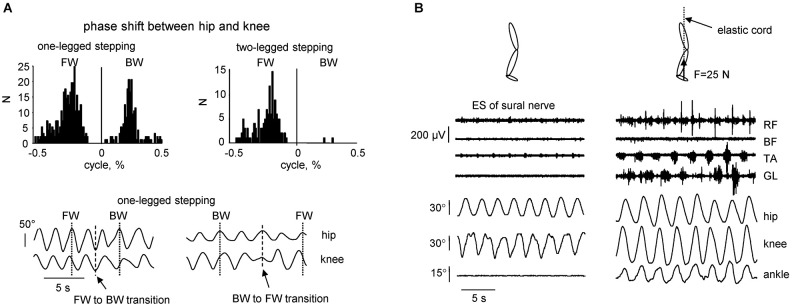
**Kinematic features of non-voluntary air-stepping movements. (A)** one-legged vs. two-legged air-stepping evoked by quadriceps muscle vibration. Upper panels—histogram of the phase shift between hip and knee joints across subjects and probes. Note similar occurrence of forward and backward one-legged air-stepping and predominantly forward 2-legged stepping. Low panels—examples of transitions (in the middle of the record) from FW to BW stepping and vice versa in 2 subjects. **(B)** examples of rhythmic leg movements evoked by continuous electrical stimulation (ES) of the sural nerve in the absence (left) and presence (right) of small (25 N) force applied to the forefoot part of the foot. The force was applied approximately in the direction of the longitudinal axis of the body using a long elastic thread cord. The length of the thread cord was about 5 m so that fluctuations in its force due to the length changes were minimal (<10%) during air-stepping. Eight consecutive cycles are shown for each condition. Note the appearance of noticeable oscillations in the ankle joint angle and activity in the distal muscles in the presence of small load force (adapted from Selionov et al., 2009).

**Figure 3:**
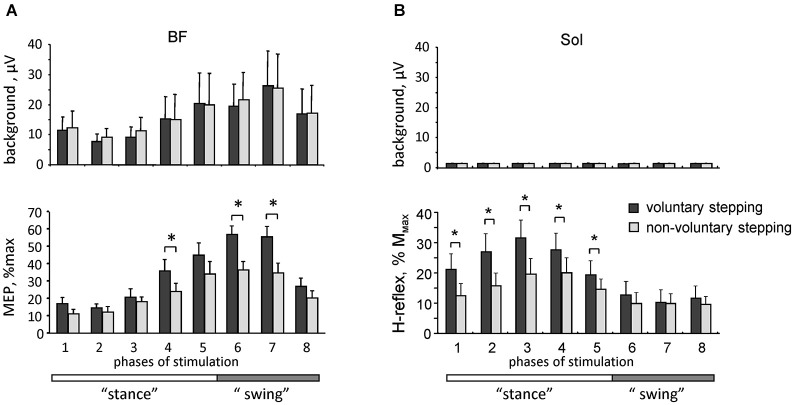
**Motor responses during voluntary and non-voluntary air-stepping in healthy subjects. (A)** background EMG activity (upper panel) and motor evoked potentials (lower panel) in response to transcranial magnetic stimulation of the motor cortex (MEPs, mean ± SE, *n* = 8 subjects) in the BF muscle during different phases of the step cycle. **(B)** background soleus EMG activity (upper panel) and H-reflex (lower panel) modulation. Asterisks denote significant differences. Note facilitation of motor responses during voluntary stepping. Adapted from Solopova et al. (2014).

**Figure 4:**
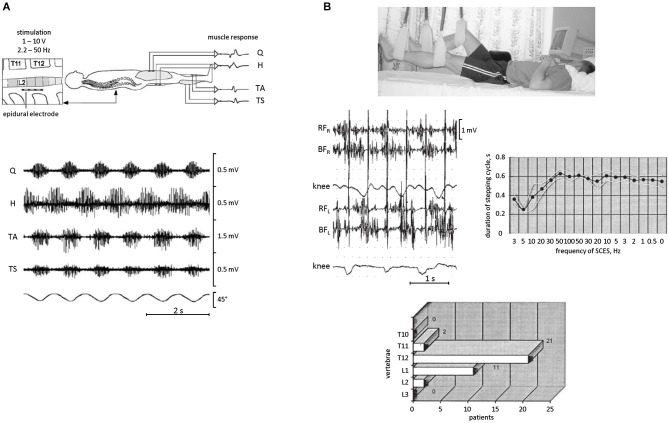
**EMG activity and rhythmic leg movements induced by epidural spinal cord electrical stimulation (SCES) in SCI patients in a supine position. (A)** epidural SCES (upper panel) and an example of EMG recordings (bottom panel) obtained from quadriceps (Q), hamstrings (H), tibialis anterior (TA), and triceps surae (TS) during SCES at 31 Hz. The goniometer traces of the knee joint angle illustrate the corresponding induced rhythmical movements of the lower limbs. Adapted from Minassian et al. (2004). **(B)** SCES-induced rhythmic leg movements in SCI patients. During SCES, the patient was lying supine and the legs were suspended on elastic straps in a position such that the hip and knee joints were in semi-flexion (top panel). Middle panels: an example of stepping-like movements at ~1 Hz evoked with 2 Hz SCES in one SCI patient. On the right—duration of stepping cycle in relationship to the frequency of SCES in this patient. The frequency gradually increased from 3 to 100 Hz and then decreased from 100 to 0.5 Hz. Bottom panel: location of the effective zone for initiating alternating stepping-like movements with SCES in a group of paraplegic patients (*n* = 29). Adapted from Shapkova (2004).

Finally, automatic, alternating movements of the legs can be initiated by upper limb movements by asking participants to move their arms rhythmically, as in hand-walking (Figure 1B; Sylos-Labini et al., 2014a). The idea is grounded on the evidence that the coordination between arms and legs during human locomotion shares many features with that in quadrupeds (Falgairolle et al., 2006; Zehr et al., 2007; Patrick et al., 2009; Dietz, 2011; Kuhtz-Buschbeck and Jing, 2012). For instance, inter-limb coupling in humans has previously been demonstrated by evoking reflexes in one limb and observing the extent to which the movement of another limb modulates reflex expression during walking (Haridas and Zehr, 2003; Mezzarane et al., 2011; Massaad et al., 2014). The coupling between the activity of cervical motoneurons underlying hand-walking and the activity of lumbosacral motoneurons underlying leg movements (Figure 1B) is presumably indirect, delayed and asynchronous (e.g., leg stepping is often characterized by a non-integer ratio between arm and leg movements frequency). These variable features suggest that signals related to arm movements do not directly entrain the motor commands to leg muscles, but affect the state of the lumbosacral locomotor circuitry, consistent with a facilitatory effect of arm swinging on cyclic leg muscle activity (de Kam et al., 2013). In addition, it has been recently shown that cervical transcutaneous stimulation of the spinal cord significantly facilitates non-voluntary air-stepping leg movements and the lumbosacral locomotor-related neuronal circuitry (Gerasimenko et al., 2014). One possible route for these trigger signals is through the intrinsic spinal pathways (propriospinal interneurons) linking cervical to lumbosacral regions in humans (Nathan et al., 1996). However, considering the latency of the leg responses relative to arm oscillations, supraspinal contributions cannot be excluded. Rhythmic arm movements imitating those during running or walking can also evoke prominent modulation of leg muscle EMGs during standing (Danna-Dos-Santos et al., 2009). Whatever the exact mechanism, these findings (Figure 1B) reinforce the idea that there exists a functional coupling between arm and leg CPGs.

## Interaction between rhythm-generation activity and sensory input

The previous studies, which aimed to activate the CPG circuits using the “air-stepping” paradigm (Gurfinkel et al., 1998; Selionov et al., 2009; Gerasimenko et al., 2010, 2014; Sylos-Labini et al., 2014a), also revealed some essential features of the intrinsic rhythm generation in humans. The evoked cyclic movements share many of their characteristics with animals. For instance, given the extensive evidence for the presence of commissural interneurons driving the contralateral locomotor circuitry (Kiehn, 2011), oscillator mechanisms and tonic influences may not be limb-specific. We found, for example, that treating one limb (e.g., applying electrical stimulation of the peroneal or sural nerves of one leg) can have its output transferred to another limb, even if the treated limb is kept stationary (Selionov et al., 2009). Also, although pattern generators for each limb have the potential to produce relatively autonomous rhythmic patterns (Forssberg et al., 1980; Yang et al., 2005), right and left sides are strongly coupled under most natural conditions (Orlovsky et al., 1999; Ivanenko et al., 2006a; Maclellan et al., 2014). Further evidence of the importance of bilateral coupling is demonstrated by the finding that two-legged stepping was more stable (and predominantly forward, Figure 2A, upper panels), whereas one-legged stepping in some subjects displayed frequent spontaneous transitions from forward to backward direction and vice versa (Figure 2A, lower panels).

Air-stepping tends to involve prominent movements in the hip and knee joints, whereas the ankle joint is typically not involved, unless minimal loading forces are applied to the foot (Figure 2B). The facilitatory effect of forces is often accompanied by modulation of the EMG activity, consistent with phase-dependent contribution of sensory activity to the pre-programmed motoneuronal drive of the distal muscles during human walking (Duysens et al., 2000; Nielsen and Sinkjaer, 2002). Even individuals with clinically motor complete paralysis demonstrate modulated activity of distal leg muscles during assisted stepping with body weight support (during locomotion with 100% body unloading, no EMG activity was present) (Harkema et al., 1997; Dietz et al., 2002). It can be concluded that afferent input from load-related receptors (including Golgi tendon organs, spindles, cutaneous receptors, and various load mechanoreceptors in the foot arch, Duysens et al., 2000; Pearson, 2004; Gravano et al., 2011) contributes to the generation of locomotor activity in the isolated human spinal cord. Therefore, the sacral pattern generation circuitry (Cazalets and Bertrand, 2000) might be inactivated when the input from the support surface is lacking. The more direct stimulation of the spinal cord locomotor circuitry using repetitive electromagnetic stimuli can evoke ankle joint oscillations (Gerasimenko et al., 2010). However, in this case it likely involves stimulation of the dorsal roots, and thus load-related afferents. Overall, the lack of ankle joint movements during non-voluntary air-stepping (Figures 1A, 2B) supports the hypothesis that the upper lumbar pattern generator activity may constitute the major oscillator “pacemaker,” whereas the sacral generator could play a subordinator role for adaptation to specific foot-support interactions. Also, minimal contact forces during air-stepping may significantly improve accurate foot trajectory control, suggesting that the support surface represents an importance reference frame and is included in the locomotor body scheme (Ivanenko et al., 2002).

## Engagement of supraspinal motor areas

Better understanding of interactions between spinal and supraspinal influences on the state of CPG circuitry may be important for developing gait rehabilitation strategies in individuals with spinal cord and brain injuries. In addition, there is an increasing consensus that motor centers in the brain, and the motor cortex in particular, play an essential and greater role in human walking compared to other mammals (Capaday, 2002; Yang and Gorassini, 2006; Petersen et al., 2012; Beloozerova et al., 2013). For instance, the coherence analysis demonstrated significant coupling between EEG recordings over the leg motor area and EMG from the tibialis anterior muscle prior to heel strike during the swing phase of walking, suggesting that the motor cortex and corticospinal tract contribute directly to the muscle activity observed in steady-state human walking (Petersen et al., 2012). Recently, we compared motor evoked potentials (MEP) in response to transcranial magnetic stimulation of the motor cortex and the H-reflex during voluntary and vibration-induced air-stepping movements in healthy humans (Solopova et al., 2014). Both the MEPs and H-reflex were significantly smaller during vibration-induced cyclic leg movements at matched amplitudes of angular motion and muscle activity (Figure 3). One may suppose that in both cases the locomotor-like leg movements are evoked via activation of the spinal pattern generation circuitry. The greater responsiveness to central inputs during voluntary CPG activation (Figure 3) may be related to facilitation of transcortical reflex pathways (Christensen et al., 1999), increased depolarization of motoneurons, and/or an overall facilitatory effect on spinal motoneurons and interneurons. Interestingly, modulation of the H-reflex was observed in the absence of noticeable background EMG activity of the soleus and tibialis anterior muscles (likely due to the absence of limb loading and ankle joint movements), and occurred during the hypothetical stance phase of the step cycle (Figure 3), consistent with a CPG phase-related modulation of spinal reflexes.

These findings highlight differences between voluntary and non-voluntary activation of the spinal pattern generator circuitry, presumably due to an extra facilitatory effect of voluntary control of stepping on spinal motoneurons and interneurons. It has been argued that the engagement of supraspinal motor areas may be beneficial for gait recovery (van den Brand et al., 2012), and there is a link between facilitation of segmental reflexes and the ability to recover gait (Dietz et al., 2009; Thompson and Wolpaw, 2014). Our results (Figure 3) support this hypothesis, and show an overall facilitatory effect of supraspinally mediated stepping on reflex responses. Such investigations may contribute to the clinical development of CPG-modulating therapies (Guertin, 2014).

## Tapping into rhythm generation circuitry in neuromotor disorders

During the last decade, there has been a growing interest in understanding an appropriate state of the spinal circuitry for performing locomotor movements (Hultborn, 2001; Edgerton et al., 2008; van den Brand et al., 2012; Selionov et al., 2013). In particular, to trigger the CPG by neurons with command function (Grillner, 2006), the physiological state of the spinal network needs to be properly prepared (Edgerton et al., 2008) since the same interneurons and motoneurons may contribute to a vast repertoire of motor actions (Hultborn, 2001).

A facilitatory effect of simulated weightlessness can be used for investigating rhythmogenesis of the spinal cord in injured populations and for entraining the spinal locomotor circuitry. Epidural stimulation is a technique that has been used for a number of years to treat individuals with a spinal cord injury, and various experiments emphasized a significant complementary effect of epidural stimulation when combined with pharmacological facilitation, e.g., serotonergic agonists, and step training (Shapkova and Schomburg, 2001; Minassian et al., 2007; Gerasimenko et al., 2008). The existence of a spinal locomotor generator circuitry in humans has been confirmed based on observations in patients with a severe spinal cord injury implanted with an array of electrodes directly placed over the back portion of the lower thoracic-upper lumbar spinal cord (Minassian et al., 2004; Shapkova, 2004). In these experiments, stepping-like movements were produced in patients who were supine with the legs in the resting position (Figure 4A) or suspended in the air (Figure 4B). Epidural stimulation could even produce rhythmic EMG activities without step-related sensory feedback (stationary legs) or with a rhythm frequency independent of that of passive treadmill stepping (Minassian et al., 2013). Nevertheless, leg suspension significantly facilitates the manifestation of rhythmic motion (Figure 4B) and permits to reveal its characteristics. For instance, depending on the exact location of the stimulating electrodes, the stimulation could produce different patterns of rhythmic leg movements with different involvements of leg joints (Shapkova, 2004), consistent with the idea that there exist individual CPGs for each limb and/or each segment, and are coordinated during natural locomotion to produce a coherent interlimb pattern (Graham Brown, 1912; Grillner, 1981). Epidural stimulation can also transform the CPG circuitry into the active functional state which persists even after a significant decrease of stimulation frequency (Figure 4B, right panel). Interestingly, non-voluntary (evoked by epidural stimulation) air-stepping movements in incomplete spinal cord injury individuals can be sustained for more than 1 h with increasing EMG activity, while voluntarily initiated rhythmic leg movements in these patients demonstrate progressive fatigue after several minutes (Shapkova, 2004). Thus, even though supraspinally mediated activation of stepping has an overall facilitatory effect on reflex responses (Figure 3) and pattern generation (Solopova et al., 2014), it may also contribute to the development of “central” fatigue (Taylor et al., 2006). Furthermore, daily sessions with epidural stimulation evoking air-stepping rhythmic movements were effective in restoring the locomotor function in some children with a severe spinal cord injury (Shapkova, 2004).

The residual sensory pathways may be critical in regaining voluntary movement. Moreover, the neuromodulation and activation of the “locomotor state” of the spinal circuitry below the lesion may enable completely paralyzed individuals to process conceptual, auditory and visual inputs, and to regain some voluntary control of paralyzed muscles (Angeli et al., 2014). In this study, a stimulation protocol was developed to allow the individuals to stimulate for ~1 h while practicing intentional movement in the supine position. Four individuals diagnosed with clinically motor complete paralysis (classified as AIS-B and AIS-A before implantation) and implanted with a lumbrosacral spinal cord stimulator at least 2.2 years post injury were able to generate EMG activity and movement during ankle dorsiflexion in the presence of epidural stimulation following a verbal command. No motor activity was present when attempting to move without epidural stimulation. Furthermore, daily training resulted in the generation of voluntary efforts with higher forces and lower stimulation voltages to reach the thresholds that enabled voluntary motor responses that could be modulated by visual and/or auditory input (Angeli et al., 2014). Hence, it is essential to discern how the spinal pattern generation circuitry is controlled by sensory input and supraspinal networks to design new rehabilitation devices that involve modulation of the physiological state of the spinal cord during training. A degradation of spinal neuronal activity takes place following a spinal cord injury, suggesting that a continuous training approach starting early after injury is necessary to maintain neuronal activity below the level of the lesion (Dietz and Müller, 2004). Future studies may focus on the mechanisms underlying the manifestation of early motor symptoms, muscle tone, impaired sensory feedback and their relation to rhythmogenesis investigated under simulated weightlessness conditions. This may also help facilitating the application of neurophysiological analyses as quantification tools for evaluating new medications useful to assess or augment the rhythmogenesis capacity and gait recovery in neurological disorders.

## Concluding remarks

Novel pharmacological strategies (Roy et al., 2012; Borton et al., 2014; Guertin, 2014) and electromagnetic stimulation techniques (Shapkova and Schomburg, 2001; Minassian et al., 2007; Gerasimenko et al., 2008; Selionov et al., 2009; Angeli et al., 2014) are being developed aimed at modulating spinal activity and restoring the locomotor function. Even though electrochemical or sensory stimulations do not necessarily induce automated stepping by activating CPG networks, they may transform lumbosacral circuits from non-functional to functional states, enabling the information-processing interface in the spinal cord to utilize multifaceted sensory input as a source of control for locomotion (Courtine et al., 2009). Overall, recent findings highlight the importance of investigating the tonic “state” of the spinal circuits. Since the air-stepping is free from many of the mechanical constraints of normal walking, it may provide an effective model for studying how peripheral inputs influence CPG behavior in human adults (Gurfinkel et al., 1998; Shapkova and Schomburg, 2001; Selionov et al., 2009; Gerasimenko et al., 2010; Solopova et al., 2014; Sylos-Labini et al., 2014a). Thus, the beneficial effect of simulated weightlessness on rhythmogenesis may enhance the utility of spinal cord stimulation techniques for developing CPG-modulating therapies and augmentation of function for disabled people.

## Conflict of interest statement

The authors declare that the research was conducted in the absence of any commercial or financial relationships that could be construed as a potential conflict of interest.

## References

[B1] Angeli C. A. Edgerton V. R. Gerasimenko Y. P. Harkema S. J. (2014). Altering spinal cord excitability enables voluntary movements after chronic complete paralysis in humans. Brain 137, 1394–1409. 10.1093/brain/awu038 24713270 PMC3999714

[B2] Beloozerova I. N. Stout E. E. Sirota M. G. (2013). Distinct Thalamo-cortical controls for shoulder, elbow and wrist during locomotion. Front. Comput. Neurosci. 7:62. 10.3389/fncom.2013.00062 23734124 PMC3659318

[B3] Beritoff J. S. (1915). On the mode of originating of labyrinthine and cervical tonic reflexes and on their part in the reflex reactions of decerebrate preparation. Q. J. Exp. Physiol. 6, 199–229 10.1113/expphysiol.1915.sp000204

[B4] Borton D. Bonizzato M. Beauparlant J. DiGiovanna J. Moraud E. M. Wenger N.. (2014). Corticospinal neuroprostheses to restore locomotion after spinal cord injury. Neurosci. Res. 78, 21–29. 10.1016/j.neures.2013.10.001 24135130

[B5] Bussel B. Roby-Brami A. Néris O. R. Yakovleff A. (1996). Evidence for a spinal stepping generator in man. Electrophysiological study. Acta Neurobiol. Exp. (Wars) 56, 465–468. 8787207 10.55782/ane-1996-1149

[B6] Capaday C. (2002). The special nature of human walking and its neural control. Trends Neurosci. 25, 370–376. 10.1016/s0166-2236(02)02173-2 12079766

[B7] Cazalets J. R. Bertrand S. (2000). Coupling between lumbar and sacral motor networks in the neonatal rat spinal cord. Eur. J. Neurosci. 12, 2993–3002. 10.1046/j.1460-9568.2000.00169.x 10971640

[B8] Christensen L. O. Morita H. Petersen N. Nielsen J. (1999). Evidence suggesting that a transcortical reflex pathway contributes to cutaneous reflexes in the tibialis anterior muscle during walking in man. Exp. Brain Res. 124, 59–68. 10.1007/s002210050600 9928790

[B9] Courtine G. Gerasimenko Y. van den Brand R. Yew A. Musienko P. Zhong H.. (2009). Transformation of nonfunctional spinal circuits into functional states after the loss of brain input. Nat. Neurosci. 12, 1333–1342. 10.1038/nn.2401 19767747 PMC2828944

[B10] Craske B. Craske J. D. (1986). Oscillator mechanisms in the human motor system: investigating their properties using the aftercontraction effect. J. Mot. Behav. 18, 117–145. 10.1080/00222895.1986.10735374 15136276

[B11] Danna-Dos-Santos A. Shapkova E. Y. Shapkova A. L. Degani A. M. Latash M. L. (2009). Postural control during upper body locomotor-like movements: similar synergies based on dissimilar muscle modes. Exp. Brain Res. 193, 565–579. 10.1007/s00221-008-1659-3 19066871 PMC2649975

[B12] de Kam D. Rijken H. Manintveld T. Nienhuis B. Dietz V. Duysens J. (2013). Arm movements can increase leg muscle activity during submaximal recumbent stepping in neurologically intact individuals. J. Appl. Physiol. (1985) 115, 34–42. 10.1152/japplphysiol.00510.2012 23661622

[B13] Dietz V. (2009). Body weight supported gait training: from laboratory to clinical setting. Brain Res. Bull. 78, I–VI. 10.1016/s0361-9230(08)00410-3 19070780

[B14] Dietz V. (2011). Quadrupedal coordination of bipedal gait: implications for movement disorders. J. Neurol. 258, 1406–1412. 10.1007/s00415-011-6063-4 21553270

[B15] Dietz V. Grillner S. Trepp A. Hubli M. Bolliger M. (2009). Changes in spinal reflex and locomotor activity after a complete spinal cord injury: a common mechanism? Brain 132, 2196–2205. 10.1093/brain/awp124 19460795

[B16] Dietz V. Müller R. (2004). Degradation of neuronal function following a spinal cord injury: mechanisms and countermeasures. Brain 127, 2221–2231. 10.1093/brain/awh255 15269117

[B17] Dietz V. Müller R. Colombo G. (2002). Locomotor activity in spinal man: significance of afferent input from joint and load receptors. Brain J. Neurol. 125, 2626–2634. 10.1093/brain/awf273 12429590

[B18] Dominici N. Ivanenko Y. P. Cappellini G. d’Avella A. Mondì V. Cicchese M.. (2011). Locomotor primitives in newborn babies and their development. Science 334, 997–999. 10.1126/science.1210617 22096202

[B19] Duclos C. Roll R. Kavounoudias A. Roll J. P. (2004). Long-lasting body leanings following neck muscle isometric contractions. Exp. Brain Res. 158, 58–66. 10.1007/s00221-004-1871-8 15042261

[B20] Duysens J. Clarac F. Cruse H. (2000). Load-regulating mechanisms in gait and posture: comparative aspects. Physiol. Rev. 80, 83–133. 10617766 10.1152/physrev.2000.80.1.83

[B21] Edgerton V. R. Courtine G. Gerasimenko Y. P. Lavrov I. Ichiyama R. M. Fong A. J.. (2008). Training locomotor networks. Brain Res. Rev. 57, 241–254. 10.1016/j.brainresrev.2007.09.002 18022244 PMC2288528

[B22] Falgairolle M. de Seze M. Juvin L. Morin D. Cazalets J.-R. (2006). Coordinated network functioning in the spinal cord: an evolutionary perspective. J. Physiol. Paris 100, 304–316. 10.1016/j.jphysparis.2007.05.003 17658245

[B23] Forssberg H. Grillner S. Halbertsma J. (1980). The locomotion of the low spinal cat. I. Coordination within a hindlimb. Acta Physiol. Scand. 108, 269–281. 10.1111/j.1748-1716.1980.tb06533.x 7376922

[B24] Gerasimenko Y. Gorodnichev R. Machueva E. Pivovarova E. Semyenov D. Savochin A.. (2010). Novel and direct access to the human locomotor spinal circuitry. J. Neurosci. 30, 3700–3708. 10.1523/JNEUROSCI.4751-09.2010 20220003 PMC2847395

[B25] Gerasimenko Y. P. Gorodnichev R. Puhov A. Moshonkina T. Savochin A. Selionov V. A.. (2014). Initiation and modulation of locomotor circuitry output with multi-site transcutaneous electrical stimulation of the spinal cord in non-injured humans. J. Neurophysiol. [Epub ahead of print]. 10.1152/jn.00609.2014 25376784

[B26] Gerasimenko Y. Roy R. R. Edgerton V. R. (2008). Epidural stimulation: comparison of the spinal circuits that generate and control locomotion in rats, cats and humans. Exp. Neurol. 209, 417–425. 10.1016/j.expneurol.2007.07.015 17850791 PMC2288525

[B27] Goltz F. Freusberg A. (1874). Uber die Funktionen des Lendenmarkes des Hundes. Pflugers Physiol. 8, 460–498 10.1007/BF01612308

[B28] Gorodnichev R. M. Pivovarova E. A. Pukhov A. Moiseev S. A. Savokhin A. A. Moshonkina T. R.. (2012). Transcutaneous electrical stimulation of the spinal cord: non-invasive tool for activation of locomotor circuitry in human. Fiziol. Cheloveka 38, 46–56. 22679796

[B29] Graham Brown T. (1912). The factors in rhythmic activity of the nervous system. Proc. R. Soc. B Biol. Sci. 85, 278–289 10.1098/rspb.1912.0051

[B30] Grasso R. Ivanenko Y. P. Zago M. Molinari M. Scivoletto G. Castellano V.. (2004). Distributed plasticity of locomotor pattern generators in spinal cord injured patients. Brain 127, 1019–1034. 10.1093/brain/awh115 14988161

[B31] Gravano S. Ivanenko Y. P. Maccioni G. Macellari V. Poppele R. E. Lacquaniti F. (2011). A novel approach to mechanical foot stimulation during human locomotion under body weight support. Hum. Mov. Sci. 30, 352–367. 10.1016/j.humov.2010.01.002 20417979

[B32] Grillner S. (1981). “Control of locomotion in bipeds, tetrapods and fish,” in Handbook of Physiology: Section 1: The Nervous System, volume II, Part.1 Motor Control, eds Brooks V. B. Brookhart J. M. Mountcastle V. B. (Bethesda, MD: Am. Physiol. Soc.), 1179–1236.

[B33] Grillner S. (2006). Biological pattern generation: the cellular and computational logic of networks in motion. Neuron 52, 751–766. 10.1016/j.neuron.2006.11.008 17145498

[B34] Guertin P. A. (2014). Preclinical evidence supporting the clinical development of central pattern generator-modulating therapies for chronic spinal cord-injured patients. Front. Hum. Neurosci. 8:272. 10.3389/fnhum.2014.00272 24910602 PMC4038974

[B35] Gurfinkel V. S. Levik Y. S. Kazennikov O. V. Selionov V. A. (1998). Locomotor-like movements evoked by leg muscle vibration in humans. Eur. J. Neurosci. 10, 1608–1612. 10.1046/j.1460-9568.1998.00179.x 9751133

[B36] Haridas C. Zehr E. P. (2003). Coordinated interlimb compensatory responses to electrical stimulation of cutaneous nerves in the hand and foot during walking. J. Neurophysiol. 90, 2850–2861. 10.1152/jn.00531.2003 12853441

[B37] Harkema S. J. Hurley S. L. Patel U. K. Requejo P. S. Dobkin B. H. Edgerton V. R. (1997). Human lumbosacral spinal cord interprets loading during stepping. J. Neurophysiol. 77, 797–811. 9065851 10.1152/jn.1997.77.2.797

[B38] Hoogkamer W. Meyns P. Duysens J. (2014). Steps forward in understanding backward gait: from basic circuits to rehabilitation. Exerc. Sport Sci. Rev. 42, 23–29. 10.1249/JES.0000000000000000 24188982

[B39] Hubli M. Dietz V. (2013). The physiological basis of neurorehabilitation - locomotor training after spinal cord injury. J. Neuroeng. Rehabil. 10:5. 10.1186/1743-0003-10-5 23336934 PMC3584845

[B40] Hultborn H. (2001). State-dependent modulation of sensory feedback. J. Physiol. 533, 5–13. 10.1111/j.1469-7793.2001.0005b.x 11351007 PMC2278613

[B41] Ivanenko Y. P. Dominici N. Cappellini G. Di Paolo A. Giannini C. Poppele R. E.. (2013). Changes in the spinal segmental motor output for stepping during development from infant to adult. J. Neurosci. 33, 3025a–3036a. 10.1523/JNEUROSCI.2722-12.2013 23407959 PMC6619203

[B42] Ivanenko Y. P. Grasso R. Macellari V. Lacquaniti F. (2002). Control of foot trajectory in human locomotion: role of ground contact forces in simulated reduced gravity. J. Neurophysiol. 87, 3070–3089. 12037209 10.1152/jn.2002.87.6.3070

[B43] Ivanenko Y. P. Poppele R. E. Lacquaniti F. (2006a). Motor control programs and walking. Neuroscientist 12, 339–348. 10.1177/1073858406287987 16840710

[B44] Ivanenko Y. P. Wright W. G. Gurfinkel V. S. Horak F. Cordo P. (2006b). Interaction of involuntary post-contraction activity with locomotor movements. Exp. Brain Res. 169, 255–260. 10.1007/s00221-005-0324-3 16369781 PMC1363359

[B45] Jordan L. M. Liu J. Hedlund P. B. Akay T. Pearson K. G. (2008). Descending command systems for the initiation of locomotion in mammals. Brain Res. Rev. 57, 183–191. 10.1016/j.brainresrev.2007.07.019 17928060

[B46] Kiehn O. (2011). Development and functional organization of spinal locomotor circuits. Curr. Opin. Neurobiol. 21, 100–109. 10.1016/j.conb.2010.09.004 20889331

[B47] Kohnstamm O. (1915). Demonstration einer katatonieartigen Erscheinung beim Gesunden (Katatonusversuch). Neurol Zent. Bl 34S, 290–291.

[B48] Kuhtz-Buschbeck J. P. Jing B. (2012). Activity of upper limb muscles during human walking. J. Electromyogr. Kinesiol. 22, 199–206. 10.1016/j.jelekin.2011.08.014 21945656

[B49] Maclellan M. J. Ivanenko Y. P. Massaad F. Bruijn S. M. Duysens J. Lacquaniti F. (2014). Muscle activation patterns are bilaterally linked during split-belt treadmill walking in humans. J. Neurophysiol. 111, 1541–1552. 10.1152/jn.00437.2013 24478155 PMC4035776

[B50] Massaad F. Levin O. Meyns P. Drijkoningen D. Swinnen S. P. Duysens J. (2014). Arm sway holds sway: locomotor-like modulation of leg reflexes when arms swing in alternation. Neuroscience 258, 34–46. 10.1016/j.neuroscience.2013.10.007 24144625

[B51] Meyns P. Van de Crommert H. W. A. A. Rijken H. van Kuppevelt D. H. J. M. Duysens J. (2014). Locomotor training with body weight support in SCI: EMG improvement is more optimally expressed at a low testing speed. Spinal Cord 52, 887–893. 10.1038/sc.2014.172 25311847

[B52] Mezzarane R. Klimstra M. Lewis A. Hundza S. Zehr E. (2011). Interlimb coupling from the arms to legs is differentially specified for populations of motor units comprising the compound H-reflex during “reduced” human locomotion. Exp. Brain Res. 208, 157–168. 10.1007/s00221-010-2467-0 21063693

[B53] Minassian K. Hofstoetter U. S. Danner S. M. Mayr W. McKay W. B. Tansey K. (2013). Mechanisms of rhythm generation of the human lumbar spinal cord in response to tonic stimulation without and with step-related sensory feedback. Biomed. Eng. Biomed. Tech. Available online at: http://www.degruyter.com/view/j/bmte.2013.58.issue-s1-A/bmt-2013-4013/bmt-2013-4013.xml;jsessionid=AF92C08F8D31046DE80E9B1BB68DC420. Accessed on December 15, 2014. 10.1515/bmt-2013-4013 24042620

[B54] Minassian K. Jilge B. Rattay F. Pinter M. M. Binder H. Gerstenbrand F.. (2004). Stepping-like movements in humans with complete spinal cord injury induced by epidural stimulation of the lumbar cord: electromyographic study of compound muscle action potentials. Spinal Cord 42, 401–416. 10.1038/sj.sc.3101615 15124000

[B55] Minassian K. Persy I. Rattay F. Pinter M. M. Kern H. Dimitrijevic M. R. (2007). Human lumbar cord circuitries can be activated by extrinsic tonic input to generate locomotor-like activity. Hum. Mov. Sci. 26, 275–295. 10.1016/j.humov.2007.01.005 17343947

[B56] Moraru E. Onose G. (2014). Current issues and considerations about the central role of rehabilitation therapies in the functional recovery of neurological impairments after stroke in adults. J. Med. Life 7, 368–372. 25408756 PMC4233440

[B57] Mori S. Kawahara K. Sakamoto T. Aoki M. Tomiyama T. (1982). Setting and resetting of level of postural muscle tone in decerebrate cat by stimulation of brain stem. J. Neurophysiol. 48, 737–748. 7131051 10.1152/jn.1982.48.3.737

[B58] Nathan P. W. Smith M. Deacon P. (1996). Vestibulospinal, reticulospinal and descending propriospinal nerve fibres in man. Brain 119, 1809–1833. 10.1093/brain/119.6.1809 9009990

[B59] Nielsen J. B. Sinkjaer T. (2002). Afferent feedback in the control of human gait. J. Electromyogr. Kinesiol. 12, 213–217. 10.1016/s1050-6411(02)00023-8 12086816

[B60] Orlovsky G. N. Deliagina T. G. Grillner S. Orlovskii G. N. Grillner S. (1999). Neuronal Control of Locomotion: From Mollusc to Man. Oxford, UK: Oxford University Press.

[B61] Patrick S. K. Noah J. A. Yang J. F. (2009). Interlimb coordination in human crawling reveals similarities in development and neural control with quadrupeds. J. Neurophysiol. 101, 603–613. 10.1152/jn.91125.2008 19036860 PMC2657078

[B62] Pearson K. G. (2004). Generating the walking gait: role of sensory feedback. Prog. Brain Res. 143, 123–129. 10.1016/s0079-6123(03)43012-4 14653157

[B63] Petersen T. H. Willerslev-Olsen M. Conway B. A. Nielsen J. B. (2012). The motor cortex drives the muscles during walking in human subjects. J. Physiol. 590, 2443–2452. 10.1113/jphysiol.2012.227397 22393252 PMC3424763

[B64] Picelli A. Melotti C. Origano F. Neri R. Waldner A. Smania N. (2013). Robot-assisted gait training versus equal intensity treadmill training in patients with mild to moderate Parkinson’s disease: a randomized controlled trial. Parkinsonism Relat. Disord. 19, 605–610. 10.1016/j.parkreldis.2013.02.010 23490463

[B65] Roy R. R. Harkema S. J. Edgerton V. R. (2012). Basic concepts of activity-based interventions for improved recovery of motor function after spinal cord injury. Arch. Phys. Med. Rehabil. 93, 1487–1497. 10.1016/j.apmr.2012.04.034 22920448

[B66] Sale P. Franceschini M. Waldner A. Hesse S. (2012). Use of the robot assisted gait therapy in rehabilitation of patients with stroke and spinal cord injury. Eur. J. Phys. Rehabil. Med. 48, 111–121. 22543557

[B67] Scivoletto G. Tamburella F. Laurenza L. Torre M. Molinari M. (2014). Who is going to walk? A review of the factors influencing walking recovery after spinal cord injury. Front. Hum. Neurosci. 8:141. 10.3389/fnhum.2014.00141 24659962 PMC3952432

[B68] Selionov V. A. Ivanenko Y. P. Solopova I. A. Gurfinkel V. S. (2009). Tonic central and sensory stimuli facilitate involuntary air-stepping in humans. J. Neurophysiol. 101, 2847–2858. 10.1152/jn.90895.2008 19339461

[B69] Selionov V. A. Solopova I. A. Zhvansky D. S. Karabanov A. V. Chernikova L. A. Gurfinkel V. S.. (2013). Lack of non-voluntary stepping responses in Parkinson’s disease. Neuroscience 235, 96–108. 10.1016/j.neuroscience.2012.12.064 23321538

[B70] Shapkova E. Y. (2004). “Spinal locomotor capabality revealed by electrical stimulation of the lumbar enlargement in paraplegic patients,” in Progress in Motor Control, eds Latash M. Levin M. (Champaign, IL: Human Kinetics), 253–289.

[B71] Shapkova E. Y. Schomburg E. D. (2001). Two types of motor modulation underlying human stepping evoked by spinal cord electrical stimulation (SCES). Acta Physiol. Pharmacol. Bulg. 26, 155–157. 11695529

[B72] Sherrington C. S. (1910). Flexion-reflex of the limb, crossed extension-reflex and reflex stepping and standing. J. Physiol. 40, 28–121. 10.1113/jphysiol.1910.sp001362 16993027 PMC1533734

[B73] Shik M. L. (1997). Recognizing propriospinal and reticulospinal systems of initiation of stepping. Motor Control 1, 310–313.

[B74] Shik M. L. Severin F. V. Orlovskiĭ G. N. (1966). Control of walking and running by means of electric stimulation of the midbrain. Biofizika 11, 659–666. 6000625

[B75] Solopova I. A. Selionov V. A. Kazennikov O. V. Ivanenko Y. P. (2014). Effects of transcranial magnetic stimulation during voluntary and non-voluntary stepping movements in humans. Neurosci. Lett. 579, 64–69. 10.1016/j.neulet.2014.07.015 25038416

[B76] Swinnen E. Baeyens J.-P. Pintens S. Van Nieuwenhoven J. Ilsbroukx S. Buyl R.. (2014). Trunk kinematics during walking in persons with multiple sclerosis: the influence of body weight support. NeuroRehabilitation 34, 731–740. 10.3233/NRE-141089 24796441

[B77] Sylos-Labini F. Ivanenko Y. P. Maclellan M. J. Cappellini G. Poppele R. E. Lacquaniti F. (2014a). Locomotor-like leg movements evoked by rhythmic arm movements in humans. PloS One 9:e90775. 10.1371/journal.pone.0090775 24608249 PMC3946538

[B78] Sylos-Labini F. La Scaleia V. d’ Avella A. Pisotta I. Tamburella F. Scivoletto G.. (2014b). EMG patterns during assisted walking in the exoskeleton. Front. Hum. Neurosci. 8:423. 10.3389/fnhum.2014.00423 24982628 PMC4058900

[B79] Taylor J. L. Todd G. Gandevia S. C. (2006). Evidence for a supraspinal contribution to human muscle fatigue. Clin. Exp. Pharmacol. Physiol. 33, 400–405. 10.1111/j.1440-1681.2006.04363.x 16620309

[B80] Thompson A. K. Wolpaw J. R. (2014). Operant conditioning of spinal reflexes: from basic science to clinical therapy. Front. Integr. Neurosci. 8:25. 10.3389/fnint.2014.00025 24672441 PMC3957063

[B81] Valentin-Gudiol M. Bagur-Calafat C. Girabent-Farrés M. Hadders-Algra M. Mattern-Baxter K. Angulo-Barroso R. (2013). Treadmill interventions with partial body weight support in children under six years of age at risk of neuromotor delay: a report of a Cochrane systematic review and meta-analysis. Eur. J. Phys. Rehabil. Med. 49, 67–91. 23575201

[B82] van den Brand R. Heutschi J. Barraud Q. DiGiovanna J. Bartholdi K. Huerlimann M.. (2012). Restoring voluntary control of locomotion after paralyzing spinal cord injury. Science 336, 1182–1185. 10.1126/science.1217416 22654062

[B83] Wirz M. Zemon D. H. Rupp R. Scheel A. Colombo G. Dietz V.. (2005). Effectiveness of automated locomotor training in patients with chronic incomplete spinal cord injury: a multicenter trial. Arch. Phys. Med. Rehabil. 86, 672–680. 10.1016/j.apmr.2004.08.004 15827916

[B84] Yang J. F. Gorassini M. (2006). Spinal and brain control of human walking: implications for retraining of walking. Neuroscientist 12, 379–389. 10.1177/1073858406292151 16957000

[B85] Yang J. F. Lamont E. V. Pang M. Y. C. (2005). Split-belt treadmill stepping in infants suggests autonomous pattern generators for the left and right leg in humans. J. Neurosci. 25, 6869–6876. 10.1523/jneurosci.1765-05.2005 16033896 PMC6725348

[B86] Zehr E. P. (2005). Neural control of rhythmic human movement: the common core hypothesis. Exerc. Sport Sci. Rev. 33, 54–60. 15640722

[B87] Zehr E. P. Balter J. E. Ferris D. P. Hundza S. R. Loadman P. M. Stoloff R. H. (2007). Neural regulation of rhythmic arm and leg movement is conserved across human locomotor tasks. J. Physiol. 582, 209–227. 10.1113/jphysiol.2007.133843 17463036 PMC2075277

